# Determining the Protective Efficacy of Toll-Like Receptor Ligands to Minimize H9N2 Avian Influenza Virus Transmission in Chickens

**DOI:** 10.3390/v15010238

**Published:** 2023-01-14

**Authors:** Sugandha Raj, Mohammadali Alizadeh, Bahram Shoojadoost, Douglas Hodgins, Éva Nagy, Samira Mubareka, Khalil Karimi, Shahriar Behboudi, Shayan Sharif

**Affiliations:** 1 Department of Pathobiology, Ontario Veterinary College, University of Guelph, Guelph, ON N1G 2W1, Canada; 2 CEVA Animal Health, 131 Malcolm Road, Guelph, ON N1K 1A8, Canada; 3 Sunnybrook Research Institute, Department of Laboratory Medicine and Pathobiology, University of Toronto, Toronto, ON M4N 3M5, Canada; 4 The Pirbright Institute, Pirbright GU24 0NE, UK

**Keywords:** chicken, H9N2 AIV, TLR ligands, shedding, immune responses, cytokines, CpG ODN 2007, poly(I:C)

## Abstract

Low-pathogenicity avian influenza viruses (AIV) of the H9N2 subtype can infect and cause disease in chickens. Little is known about the efficacy of immune-based strategies for reducing the transmission of these viruses. The present study investigated the efficacy of Toll-like receptor (TLR) ligands (CpG ODN 2007 and poly(I:C)) to reduce H9N2 AIV transmission from TLR-treated seeder (trial 1) or inoculated chickens (trial 2) to naive chickens. The results from trial 1 revealed that a low dose of CpG ODN 2007 led to the highest reduction in oral shedding, and a high dose of poly(I:C) was effective at reducing oral and cloacal shedding. Regarding transmission, the recipient chickens exposed to CpG ODN 2007 low-dose-treated seeder chickens showed a maximum reduction in shedding with the lowest number of AIV+ chickens. The results from trial 2 revealed a maximum reduction in oral and cloacal shedding in the poly(I:C) high-dose-treated chickens (recipients), followed by the low-dose CpG ODN 2007 group. In these two groups, the expression of type I interferons (IFNs), protein kinase R (PKR), interferon-induced transmembrane protein 3 (IFITM3), viperin, and (interleukin) IL-1β, IL-8, and 1L-18 was upregulated in the spleen, cecal tonsils and lungs. Hence, TLR ligands can reduce AIV transmission in chickens.

## 1. Introduction

Avian influenza viruses (AIV) are enveloped, single-stranded, negative-sense RNA viruses with a segmented genome belonging to the family *Orthomyxoviridae*. AIV are influenza A viruses and are further subdivided into high-pathogenic avian influenza viruses (HPAIVs) and low-pathogenic AIVs (LPAIVs) [1]. Migratory waterfowl, such as geese, ducks, gulls and shorebirds, are major reservoirs for LPAIVs [2]. The H9N2 AIV subtype can be transmitted via various routes and in different avian and mammalian species, including chickens, turkeys, ducks, pigs, horses and humans [3,4,5,6]. The H9N2 AIV has the potential to spread across wide geographical distances. It is a prevalent subtype and is endemic in the Middle East, Africa, Europe and Central Asian countries [7,8]. Sporadic outbreaks in poultry associated with H9N2 AIV were reported in 2003, 2016 and 2017 in China and Hong Kong [7,9]. These outbreaks were associated with significant economic losses in the poultry sector, largely due to a decrease in egg and meat production and increased feed conversion ratios [10]. Outbreaks of H9N2 AIV in poultry can cause mild-to-subclinical respiratory and gastro-intestinal infections with fatalities associated with secondary bacterial infections [11].

Major concerns with H9N2 AIV infections are associated with the high transmission rate, which generally occurs in the form of spillovers from wild migratory birds to domestic poultry [12,13]. The transmission of H9N2 AIV is known to occur in turkeys, quail, crow, sparrows and pigs [14,15,16]; however, its transmission from avian species to humans was also well documented between 2015 and 2021 [17]. Therefore, for the following reasons, the present study sought to explore the potential of H9N2 AIV to infect chickens. Outbreaks related to H7N7, H7N9 (2012) and, more recently, H5N6 virus, have been reported in China [16,17]. This demonstrates the zoonotic potential of H9N2 AIV to infect humans, and thus, pose a public health risk.

To limit AIV transmission in poultry, biosecurity measures including isolation, quarantine, stamping-out policies and vaccination have been considered over the years [18,19,20,21,22]. Preventive vaccine strategies become a useful tool only when the subtype of AIV has been identified. However, the ability of vaccines to provide complete protection to emerging strains remains questionable. Thus, we hypothesize that immunostimulatory agents such as Toll-like receptor ligands (TLRs) could be applied as an alternative approach to restrict the transmission of LPAIVs in poultry [23,24,25].

TLRs have been widely studied in mammals and chickens as sole ligands or as adjuvants [26,27,28,29]. TLRs are a conserved group of pattern recognition receptors which sense pathogen-associated molecular patterns (PAMPs) on the pathogens and trigger host immune responses via different signaling pathways [30]. TLRs have been shown to enhance host immune responses against Marek’s disease virus [31,32] and infectious bronchitis virus [33]. Chickens express a number of TLRs including TLR1, TLR2, TLR3, TLR4, TLR5, TLR7, TLR15 and TLR21 [28]. They are diverse in their distribution on different cell subsets such as antigen-presenting cells (APCs) and epithelial cells. TLRs mediate their action as sole ligands via the production of pro-inflammatory and anti-viral cytokines against different pathogens [30,34,35]. For instance, in our previous study, we focused on employing TLR3 in chickens, as it can sense double-stranded (ds) RNA (a stage in the replication cycle of viruses) and the synthetic ligand polyinosinic:polycytidylic acid, poly(I:C) [36]. In one of our previous studies, we also highlighted the use of poly(I:C) as a sole ligand. The results demonstrated a significant reduction in oral and cloacal H9N2 AIV shedding associated with the induction of type I and II interferons (IFNs) [37]. Similarly, TLR21 in chickens recognizes double-stranded DNA, including CpG ODN 2007, a molecule that has previously been shown to enhance antibody-mediated responses as a sole ligand in chickens against H4N6 AIV [21].

Hence, the above findings raise the possibility that TLR ligands can be employed as useful immunostimulatory agents to enhance immunity and act as potential anti-viral agents in transmission models. Therefore, the current study aimed to determine the stand alone potential of CpG ODN 2007 and poly(I:C) to minimize H9N2 AIV shedding and transmission from infected to naive chickens.

## 2. Materials and Methods

### 2.1. Chickens

One-day-old specific pathogen-free (SPF) White Leghorn chickens were obtained from the Canadian Food Inspection Agency (Ottawa, ON, Canada). A total of three hundred and ninety-six chickens were housed in Horsfall units in the Research Isolation Unit at the University of Guelph. All of the experiments and procedures were approved by the University of Guelph Animal Care Committee (AUP 4203) and adhered to the regulations of the Canadian Council for Animal Care. The chickens were held in the Horsfall units for a period of 2 weeks to provide sufficient time for the immune system to fully develop and gain competence for responding to the virus or the treatments.

### 2.2. Avian Influenza Virus Propagation and Infection in Chickens

A low-pathogenicity A/TK/IT/13VIR1864-45/2013 H9N2 AIV was used for the present study. The virus was obtained from the Istituto Zooprofilattico Spermentale delle Venezie (IZSVe), Legnaro, Padua, Italy. The virus was propagated in 10-day-old embryonated chicken eggs by inoculation into the allantoic cavity and incubated for a period of 72 h at 37 °C. Seventy-two hours post-incubation, the eggs were maintained overnight at 4 °C. The allantoic fluid was then collected and centrifuged at 400× *g* for 15 min and stored at −80 °C [17]. The virus titers were quantified by titrating the virus on Madin-Darby canine kidney (MDCK) cells. The titers were based on the endpoint dilutions expressed as 50% tissue culture infectious dose (TCID_50_/mL) [38]. In the present study, virus inoculum containing 8 × 10^8^ TCID_50_ of H9N2 AIV in 250 μL was administered via a combination of ocular (50 μL/eye), intra-nasal (50 μL/nostril) and intra-tracheal routes (50 μL) to infect the inoculated seeder chickens in the two experimental designs.

### 2.3. TLR Ligands

Synthetic class B CpG ODN 2007 was procured from Invivogen (San Diego, California, USA), and poly(I:C) was purchased from Sigma-Aldrich (Catalogue no. P9582), Canada (Oakville, Ontario, Canada). The ligands were re-suspended in phosphate buffer saline (PBS, pH 7.4).

### 2.4. Experimental Design

Two independent trials were conducted to determine the potential of TLR ligands to exert inhibitory effects in H9N2 AIV transmission in a direct contact transmission model. The first trial consisted of fourteen-day-old chickens (*n* = 144), which were divided into 6 groups of 24 birds each. Each group was further sub-divided into ‘seeder’ (*n* = 16; TLR treated + inoculated) and ‘recipient’ subgroups (*n* = 8). The seeder chickens were injected intramuscularly (i.m.) in the pectoral muscle with a low (10 μg) or high (50 μg) dose of CpG ODN 2007 or a low (80 μg) or high dose (400 μg) of poly(I:C), while the control groups (PBS+ unchallenged and PBS+ challenged) received 100 μL of PBS. Eighteen hours post TLR treatment, the seeder chickens were inoculated with H9N2 AIV (except the PBS+ unchallenged group). The above-mentioned doses were selected based on our previous studies [36]. After 24 h of H9N2 AIV inoculation of the seeder chickens, the recipient (naive) chickens were co-housed in the isolators with seeder chickens up to 14 days post exposure (PE). (Supplementary Figure S1)

To identify the correlates of immunity associated with TLR treatment, another experiment (*n* = 108) was conducted in conjunction with trial 1. The TLR-treated seeder chickens (*n* = 6/group) were euthanized at 3, 8 and 18 h post TLR treatment, and spleen, cecal tonsils and lung samples were collected and stored in RNAlater^®^ (Thermofisher Scientifics, Baltics UAB, Vilnius, Lithuania). 

For the second trial, 14-day-old chickens were divided into 6 groups of 24 birds each (*n* = 144). The chickens in each group were further sub-grouped into ‘seeder’ (*n* = 16) or ‘recipient’ (*n* = 8; TLR treated) groups. The recipient chickens of each treatment group were injected i.m. in the pectoral muscle with 100μL of either low (80 μg) or high (400 μg) dose of poly(I:C), or a low (10 μg) or high (50 μg) dose of CpG ODN 2007, while the control groups received 50 μL of PBS. The seeder chickens were infected with H9N2 AIV (day 14 of age) and co-housed with the recipient chickens 24 h after TLR treatment or post infection (PI) (Supplementary Figure S2). To assess virus shedding, oral and cloacal swabs were collected on days 3, 5, 7 and 9 PI/PE.

### 2.5. Virus Isolation

To assess virus shedding, oral and cloacal swabs were collected at days 3, 5, 7 and 9 PI/PE from both seeder and recipient groups. The swab samples were collected using Puritan PurFlock Ultra, sterile flocked collection devices (15 cm) from Gilford, Maine, USA. The samples were transported in transport medium DMEM (Dulbecco’s Modified Eagle’s medium) supplemented with 0.5% BSA fraction V, 10 mL of penicillin (200 U/mL), /streptomycin (80 μg/mL) and 5 mL of gentamycin (50 μg/mL), and aliquoted in 1.5 mL centrifuge tubes to prevent any bacterial contamination. The samples were kept on ice throughout until processed. The samples were processed by vortexing for 1 min followed by low-speed centrifugation at 500× *g* for 10 min at 4 °C. Supernatant was aliquoted and stored at −80 °C. 

The virus titer was quantified by serially diluting swab samples over 70–95% confluent monolayer of MDCK cells, and incubation at 37 °C for 72 h. The titer was determined by identifying the highest dilution that showed a cytopathic effect (CPE) under the microscope and confirmed by performing a hemagglutination test with 0.5% chicken blood. The titer expressed as TCID50/mL was calculated using the method of Reed and Muench (1938) [38]. The minimal limit of detection for the assay was 2.25 log_10_ TCID_50_/mL. In cases where the virus shedding titer was below the limit of detection of the assay, a titer of 1.1 log_10_ TCID_50_/mL was arbitrarily assigned.

### 2.6. Hemagglutination Inhibition (HI) Assay

Mean antibody titers were determined using serum samples collected on days 7, and 14 PI/PE from the seeder and recipient chickens. The HI assay was performed as previously described in our study [21]. Briefly, sera were serially diluted (two-fold). Fifty μL of H9N2 AIV preparation containing 8 haemagglutinin units (HAU) was incubated for 30 min at room temperature (RT) in 96-well V bottom plates (Corning Inc., Corning, New York, USA). One-hundred μL of chicken red blood cells (RBCs) was then added at 0.5%, and the plates were further incubated for 30 min at RT. The HI titer was determined as the reciprocal of the greatest dilution to show complete inhibition of red blood cell agglutination (log_2_ scale). The minimal limit of detection of the assay was 1 log_2_ HI units, i.e., 2 HAI.

### 2.7. RNA Extraction, cDNA Synthesis and Real- Time PCR 

Total RNA extraction and cDNA synthesis were performed as previously described in our study [24]. The real-time PCR using SyBR Green was performed with diluted cDNA using the LightCycler^®^ 480 II (Roche Diagnostics, Basel, Switzerland), as previously described [19]. Primers were synthesized by Sigma-Aldrich, Oakville, Canada. The specific sequences of the primers are presented in Table 1. The relative expression of all target genes was calculated relative to the housekeeping gene ß-actin using the LightCycler^®^ 480 software.

### 2.8. Statistical Analysis

Differences in virus shedding, gene expression and antibody titers were analyzed using a one-way ANOVA, followed by Tukey’s post hoc test for multiple comparisons when the data had equal variances. When the data did not have equal variances, a Kruskal–Wallis test was performed. A gene expression analysis was performed using the LightCycler^®^ 480 II software (Roche Diagnostics, Basel, Switzerland) relative to the housekeeping gene β-actin and compared to the PBS+ challenged and PBS+ unchallenged control groups. Logarithmic transformations were performed when the error deviations did not have homogenous variance across the treatment groups. Differences were considered statistically significant when *p* < 0.05. The rate of infection was calculated based on the number of positive isolations in the swabs and were compared using Fisher’s exact test. 

## 3. Results

### 3.1. Trial 1

#### 3.1.1. Administration of TLR Ligands Reduces Oral and Cloacal Shedding in Seeder Chickens

**Figure 1:**
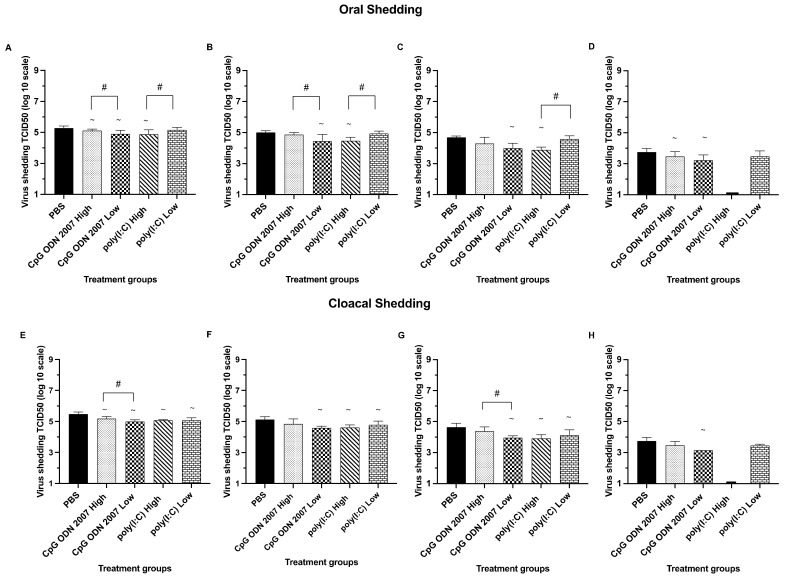
Mean virus titers in oral and cloacal swabs (trial 1) on days 3, 5, 7 and 9 post infection (PI) in the seeder (TLR treated + inoculated) chickens. Seeder chickens were treated with 100 μL of either a low (80 μg) or high (400 μg) dose of poly(I:C), or a low (10 μg) or high (50 μg) dose of CpG ODN 2007 or 100 μL of PBS for the control groups (PBS-challenged and PBS-unchallenged). Eighteen hours post TLR treatment, the seeder chickens were inoculated with H9N2 AIV (except the PBS-unchallenged control). Seeder chickens were then co-housed with recipient chickens after twenty-four hours of H9N2 AIV inoculation. Figure 1 represents the mean virus titers of H9N2 AIV (expressed in TCID_50_/mL) in oral (**A**–**D**) and cloacal swabs (**E**–**H**) on days 3, 5, 7 and 9 PI in the seeder groups, compared to the PBS-challenged group (positive control). The PBS-unchallenged control chickens remained negative in virus shedding throughout the trial and, therefore, are not used in the graphical representation. The statistical analysis was performed using a one-way ANOVA followed by Tukey’s post hoc test for multiple comparisons. When data were not normally distributed, a Kruskal–Wallis test was performed. ^~^: *p* < 0.05 (vs PBS-challenged control), ^#^: *p* < 0.05 (high dose vs low dose). In cases where the virus titer was below the limit of detection of the assay, a titer of 1.1 log_10_ TCID_50_/mL was arbitrarily assigned.

Chickens that received low and high doses of CpG ODN 2007 had a significant reduction in oral shedding (Figure 1 A–D) on day 3 PI, with mean oral shedding titers of 4.8 log_10_ TCID_50_/mL and 5.0 log_10_ TCID_50_/mL, respectively, compared to the PBS+ challenged group (5.4 log_10_ TCID_50_/mL) (*p* < 0.05). Seeder chickens that were treated with a high dose of poly(I:C) had significantly lower virus titers on days 3 (4.8 log10 TCID_50_/mL), 5 (4.4 log_10_ TCID_50_/mL) and 7 (3.8 log_10_ TCID_50_/mL), compared to the low-dose poly(I:C)-treated group at all three time points (*p* < 0.05). Additionally, the low dose of CpG ODN 2007 proved to be effective in reducing oral shedding, with an average titer of 4.8 log_10_ TCID_50_/mL on day 3 and 4.4 log_10_ TCID_50_/mL on day 5 PI when compared to the high dose of CpG ODN 2007 group (*p* < 0.05). Amongst all four treatment groups, chickens that received a high dose of poly(I:C) saw a maximum reduction in oral shedding at different time points, followed by a low dose of CpG ODN 2007, high dose of CpG ODN 2007 and low dose of poly(I:C) (*p* < 0.05). No detectable virus shedding was observed in the groups that received a high dose of poly(I:C) beyond day 7 PI.

In terms of cloacal shedding, seeder chickens treated with a high dose of poly(I:C) had reduced cloacal shedding (Figure 1E–H) on days 3 (5.0 log_10_ TCID_50_/mL), 5 (4.6 log_10_ TCID_50_/mL) and 7 PI (3.9 log_10_ TCID_50_/mL), compared to the control PBS group (*p* < 0.05). All the seeder groups had H9N2 AIV shedding up to day 9 PI; however, no detectable H9N2 AIV shedding was observed from the seeder chickens treated with a high dose of poly(I:C) beyond day 7 PI. A significant reduction in cloacal shedding was observed from the seeder chickens treated with high and low doses of CpG ODN 2007 on day 3 PI, with an average titer of 5.1 log_10_ TCID_50_/mL and 4.9 log_10_ TCID_50_/mL, respectively, compared to the PBS+ challenged group (5.5 log_10_ TCID_50_/mL) (*p* < 0.05). Chickens treated with a low dose of CpG ODN 2007 saw a maximum decline in cloacal shedding at all time points, compared to the PBS+ challenged group (*p* < 0.05). There was a significant effect in terms of dosage between seeder chickens treated with a high and low dose of CpG ODN 2007 on days 3 and 7 PI. Seeder chickens that received a low dose of CpG ODN 2007 had significantly lower cloacal shedding on days 3 (4.9 log_10_ TCID_50_/mL) and 7 PI (3.9 log_10_ TCID_50_/mL), compared to the high-dose CpG ODN 2007 group on day 3 (5.1 log_10_ TCID_50_/mL) and on day 7 (4.4 log_10_ TCID_50_/mL), respectively (*p* < 0.05). Additionally, there was a significant difference in the number of positive H9N2 AIV isolations in the low-dose-of-CpG ODN 2007 group (10/16), compared to the PBS+ challenged group, on days 3 and 7 PI, respectively (16/16) (*p* < 0.05) (Table 2).

#### 3.1.2. Poly(I:C) and CpG ODN 2007 Reduce the Transmission of H9N2 AIV in Recipient Chickens

**Figure 2:**
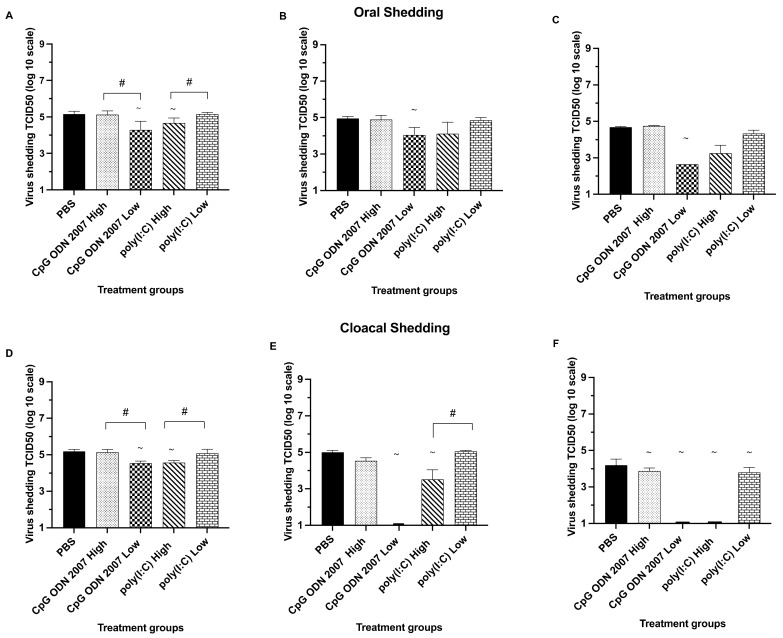
Mean virus titers in oral and cloacal swabs (trial 1) on days 3, 5, and 7 post exposure (PE) in the recipient chickens. Figure 2 represents the mean virus titers of H9N2 AIV (expressed in TCID_50_/mL) in oral (**A**–**C**) and cloacal swabs (**D**–**F**) on days 3, 5, and 7 PE in the recipient groups. The recipient chickens of the PBS-unchallenged control remained negative in virus shedding throughout the trial, and thus, are not used in the graphical representation. Seeder chickens were treated with 100 μL of either a low (80 μg) or high (400 μg) dose of poly(I:C), or a low (10 μg) or high (50 μg) dose of CpG ODN 2007 or 100 μL of PBS for the control groups (PBS-challenged and -unchallenged). Eighteen hours post TLR treatment, the seeder chickens were inoculated with H9N2 AIV (except the PBS-unchallenged control). The seeder chickens were then co-housed with recipient chickens after twenty-four hours of H9N2 AIV inoculation. The statistical analysis was performed using a one-way ANOVA followed by Tukey’s post hoc test for multiple comparisons. When data were not normally distributed, a Kruskal–Wallis test was performed. ~: *p* < 0.05 (vs PBS-challenged control), #: *p* < 0.05 (high dose vs low dose). In cases where the virus titer was below the limit of detection of the assay, a titer of 1.1 log^10^ TCID_50_/mL was arbitrarily assigned.

Regarding oral shedding (Figure 2A–C), the recipient chickens that were exposed to CpG ODN 2007 and the poly(I:C)-treated seeder chickens exhibited oral shedding up to day 7 PE, irrespective of the dosage. No detectable virus titers were observed in either of the recipient chicken groups beyond day 7 PE. Recipient chickens that were exposed to the chickens treated with a low dose of CpG ODN 2007 showed significantly lower oral shedding on days 3 (4.2 log_10_ TCID_50_/mL), 5 (4.0 log_10_ TCID_50_/mL) and 7 (2.6 log_10_ TCID_50_/mL) PE, compared to the PBS group, at all different time points (*p* < 0.05). An effect of dosage was observed between the CpG ODN 2007 groups, with the recipient chickens exposed to the seeder chickens treated with a low dose of CpG ODN 2007 showing less oral shedding on day 3 PE (4.2 log_10_ TCID_50_/mL), compared to the recipient chickens of the high-dose-of-CpG ODN 2007 group (5.2 log_10_ TCID_50_/mL) (*p* < 0.05).

Additionally, the results of virus isolation in oral swabs revealed that the recipient chickens (Table 3) exposed to the low-dose CpG ODN 2007 seeder chickens had a significantly smaller number of animals testing positive in virus isolation on day 7 PE (1/8). Similarly, a significant reduction in the isolation rate was observed in the recipient chickens exposed to the seeder chickens treated with a high dose of poly(I:C) on days 5 and 7, with 3/8 and 1/8 chickens testing positive for virus isolations, respectively (*p* < 0.05).

With respect to cloacal shedding (Figure 2D–F), all recipient groups except the high dose CpG ODN 2007 group showed a significant decline in cloacal shedding on day 7 PE compared to the PBS control group, irrespective of the dosage (*p* < 0.05). The highest reduction in cloacal shedding was observed in the recipient chickens exposed to the seeder chickens treated with a low dose of CpG ODN 2007 at different time points. Recipient chickens that were exposed to seeder chickens treated with a low dose of CpG ODN 2007 treated demonstrated a significant decrease in cloacal shedding (4.5 log_10_ TCID_50_/mL) on day 3 PE, compared to the PBS+ challenged group (5.2 log_10_ TCID_50_/mL). Interestingly, the recipient chickens of the CpG ODN 2007 low-dose group did not show any cloacal shedding beyond day 3 post exposure (*p* < 0.05). The recipient chickens of the low-dose CpG ODN 2007 group had lower H9N2 AIV titers on day 3 PE (4.5 log_10_ TCID_50_/mL), compared to the high-dose CpG ODN 2007 group (5.1 log_10_ TCID_50_/mL). Similarly, the recipient chickens exposed to the high-dose poly(I:C)-treated seeder chickens showed a significant reduction in cloacal virus shedding on days 3 (4.5 log_10_ TCID_50_/mL) and 5 PE (3.5 log_10_ TCID_50_/mL), compared to the low-dose poly(I:C) group (*p* < 0.05). There was a significant reduction in the number of positive virus isolations in the cloacal swabs obtained from the recipient chickens of the high-dose poly(I:C) group on day 5 PE with 2/8 chickens positive for virus isolation, compared to the 7/8 in the PBS group (*p* < 0.05) (Table 3).

### 3.2. Trial 2

#### 3.2.1. H9N2 AIV Infection within the Seeder Groups

All seeder chickens demonstrated oral and cloacal shedding on days 3, 5, 7 and 9 PI (Supplementary Figure S3A–D). The peak in oral shedding was observed by day 3 PI. A similar pattern of virus shedding was observed in the cloacal shedding in different seeder groups (Supplementary Figure S3E–H). A decline in mean virus shedding in the oral and cloacal swabs was evident from day 5 PI onwards up to day 9 PE in all seeder groups. Moreover, cloacal virus shedding was higher compared to oral virus load, irrespective of the time points. The data confirm the infection in seeder chickens.

#### 3.2.2. Treatment with Poly(I:C) and CpG ODN 2007 Affects the Transmission of H9N2 AIV in Recipient Chickens

Trial 2 was designed to determine the protective efficacy of TLR ligands CpG ODN 2007 and poly(I:C) in naïve recipient chickens when kept in direct contact with the H9N2-inoculated seeder chickens. 

**Figure 3:**
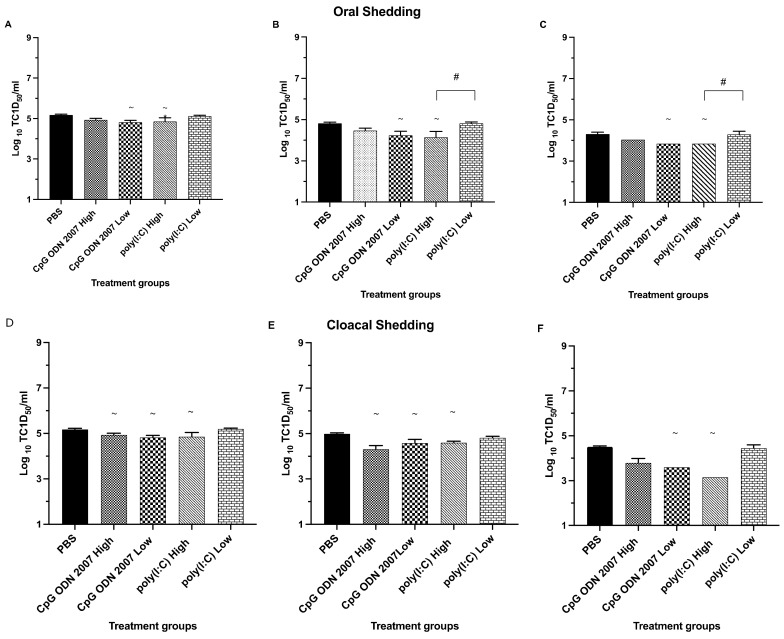
Mean virus titers in oral and cloacal swabs (trial 2) on days 3, 5, and 7 post exposure (PE) in the recipient chickens. Mean virus titers of H9N2 AIV (expressed in TCID_50_/mL) present in oral (**A**–**C**) and cloacal swabs (**D**–**F**) on days 3, 5, and 7 PE to assess virus shedding in the recipient (TLR-treated) chickens. The recipient chickens of each treatment group were injected i.m with 100 μL of either a low (80 μg) or high (400 μg) dose of poly(I:C), or a low (10 μg) or high (50 μg) dose of CpG ODN 2007, while control groups received 100 μL of PBS (PBS-challenged and PBS-unchallenged). On the other hand, the seeder chickens were inoculated with H9N2 AIV on the same day and co-housed with the recipient chickens twenty-four hours after TLR treatment/post infection. When data were not normally distributed, a Kruskal–Wallis test was performed. ^~^: *p* < 0.05 (vs PBS-challenged control), ^#^: *p* < 0.05 (high dose vs low dose).

In terms of oral shedding (Figure 3A–C), all the recipient groups treated with CpG ODN 2007 and poly(I:C) showed oral shedding up to day 7 PE. The recipient groups that were treated with the low dose of CpG ODN 2007 showed a significant reduction in virus shedding, with average shedding titers of 4.8 log_10_ TCID_50_/mL, 4.2 log_10_ TCID_50_/mL and 3.8 log_10_ TCID_50_/mL on days 3, 5 and 7 PE, compared to the PBS-treated group, with average titers of 5.2 log_10_ TCID_50_/mL, 4.8 log_10_ TCID_50_/mL and 4.4 log_10_ TCID_50_/mL, respectively (*p* < 0.05). The number of chickens orally shedding H9N2 AIV (1/8 on day 7 PE) in the low-dose CpG ODN 2007 group was significantly lower compared to the PBS-treated recipient chickens (6/8 on day 7 PE) (*p* < 0.05). Similarly, the high-dose poly(I:C)-treated recipient chickens also saw significant reductions in virus shedding on day 3 (4.7 log_10_ TCID_50_/mL) and 7 PE (3.8 log_10_ TCID_50_/mL), compared to the PBS+ challenged group, with 1/8 chickens being positive for virus isolation on day 7 PE (*p* < 0.05) (Table 4). The recipient chickens treated with a low dose of CpG ODN 2007 showed a maximum reduction in oral shedding on day 5 PE (4.2 log_10_ TCID_50_/mL). Between the high and low doses of poly(I:C)-treated recipient chickens, there was a significant difference in oral shedding on days 5 and 7 PE, with the high-dose poly(I:C)-treated group showing a higher reduction in virus shedding compared to the low-dose poly(I:C) group (*p* < 0.05).

With respect to the cloacal shedding (Figure 3D–F), the recipient chickens treated with CpG ODN 2007 (high and low) showed a significant reduction in cloacal shedding compared to the PBS control group on days 3 and 5 PE, respectively (*p* < 0.05). Moreover, the recipient chickens that were treated with the high dose of poly(I:C) also showed a significant reduction in cloacal shedding on days 3 (4.8 log_10_ TCID_50_/mL), 5 (4.6 log_10_ TCID_50_/mL) and 7 PE (3.2 log_10_ TCID_50_/mL), compared to the PBS group, which showed an average shedding of 5.2 log_10_ TCID_50_/mL, 4.9 log_10_ TCID_50_/mL and 4.5 log_10_ TCID_50_/mL on days 3, 5 and 7 PE, respectively. The number of recipient chickens with cloacal shedding was observed to be significantly reduced in the low-dose-treated CpG ODN 2007 and high-dose-poly(I:C) group, compared to the PBS group, on days 3 and 7 PE, respectively (Table 4). Amongst all the treatment groups, treatment with a high dose of poly(I:C) led to the highest reduction in cloacal shedding (3.2 log_10_ TCID_50_/mL) on day 7 PE.

#### 3.2.3. Identifying the Corelates of Immunity in the Spleen, Cecal Tonsils and Lungs in TLR Ligand Treated Chickens

##### Spleen

**Figure 4:**
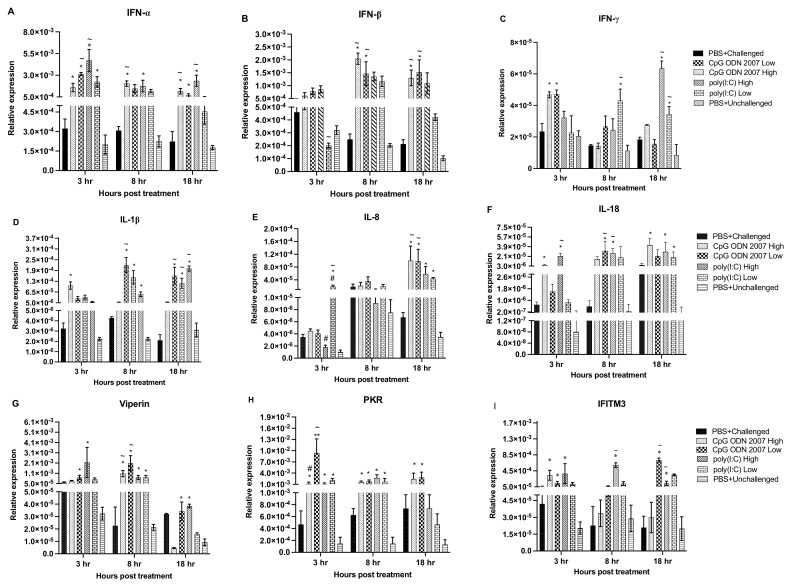
Relative gene expression in spleen at 3, 8, 18 h post CpG ODN 2007 and poly(I:C) treatment. Relative gene expression of the cytokines (**A**) IFN-α, (**B**) IFN-β and (**C**) IFN-γ, (**D**) IL-1β, (**E**) IL-8, (**F**) IL-18, ISGs- (**G**) viperin, (**H**) PKR, (**I**) IFITM3 in spleen at 3, 8, 18 h post TLR treatment. Chickens were treated with two different doses of CpG ODN 2007 (10 and 50 μg/chicken) and poly(I:C) (80 and 400 μg/chicken) or 100 μL of PBS for the control groups (PBS+ challenged or PBS+ unchallenged). The seeder chickens (*n* = 6/group) were euthanized at 3, 8 and 18 h post TLR treatment, and spleens were collected and stored in RNAlater^®^. The values represent the mean gene expression levels relative to B-actin ± standard error of the mean (SEM). Statistical significance between the group was calculated using a one-way ANOVA followed by Tukey’s multiple comparison test. The results were considered significant from PBS+ unchallenged if *p* < 0.05 * and considered statistically significant from the PBS+ challenged control group if *p* < 0.05 ^~^, in cases of significant statistical difference between two different doses of the same ligand if *p* < 0.05 ^#^.

**Figure 5:**
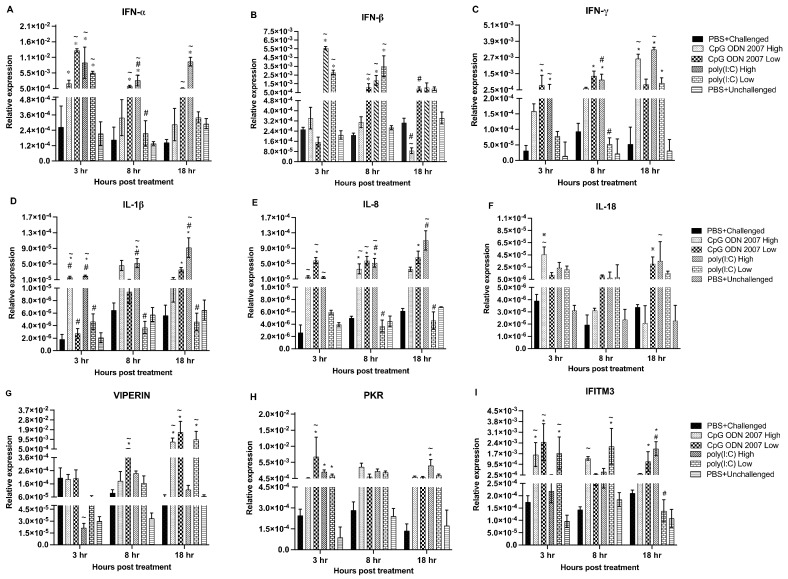
Relative gene expression in cecal tonsils at 3, 8, 18 h post CpG ODN 2007 and poly(I:C) treatment. Relative gene expression of the cytokines (**A**) IFN-α, (**B**) IFN-β and (**C**) IFN-γ, (**D**) IL-1β, (**E**) IL-8, (**F**) IL-18, ISGs- (**G**) viperin, (**H**) PKR, (**I**) IFITM3 in cecal tonsils, on 3, 8, 18 h post TLR treatment was quantified by RT-PCR. Chickens were treated with two different doses of CpG ODN 2007 (10 and 50 μg/chicken) and poly(I:C) (80 and 400 μg/chicken), or 100 μL of PBS for the control groups (PBS+ challenged or PBS+ unchallenged). The TLR-treated seeder chickens (*n* = 6/group) were euthanized at 3, 8 and 18 h post TLR treatment. The graphical values represent the mean gene expression levels relative to the B-actin + standard error of mean (SEM). Statistical significance between the groups was calculated using using a one-way ANOVA followed by Tukey’s multiple comparison test. The results were considered significant from PBS+ unchallenged if *p* < 0.05 * and considered statistically significant from the PBS+ challenged control group if *p* < 0.05 ^~^, in cases of significant statistical difference between two different doses of the same ligand if *p* < 0.05 ^#^.

**Figure 6:**
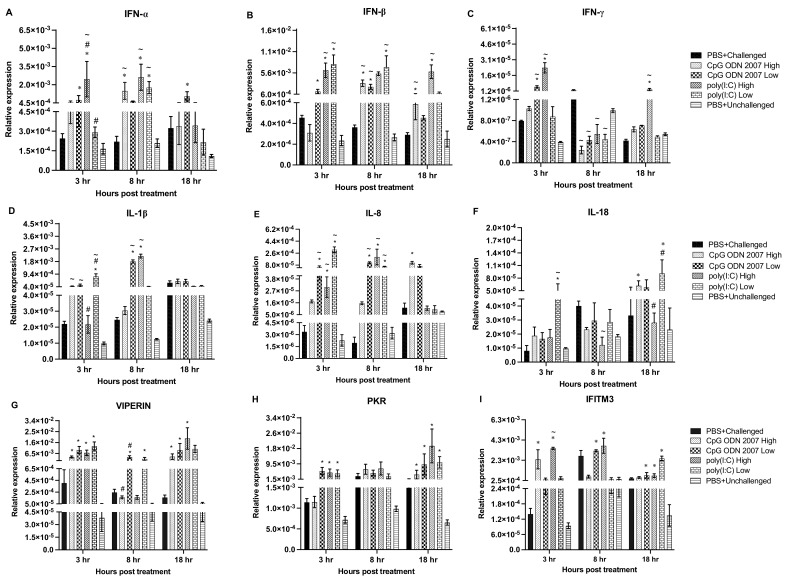
Relative gene expression in lung at 3, 8, 18 h post CpG ODN 2007 and poly(I:C) treatment. Relative gene expression of the cytokines (**A**) IFN-α, (**B**) IFN-β and (**C**) IFN-γ, (**D**) IL-1β, (**E**) IL-8, (**F**) IL-18, ISGs- (**G**) viperin, (**H**) PKR, (**I**) IFITM3 in lung, on 3, 8, 18 h post TLR treatment was quantified by RT-PCR. Chickens were treated with two different doses of CpG ODN 2007 (10 and 50 μg/chicken) and poly(I:C) (80 and 400 μg/chicken), or 100 μL of PBS for the control groups (PBS+ challenged or PBS+ unchallenged). The TLR-treated seeder chickens (*n* = 6/group) were euthanized at 3, 8 and 18 h post TLR treatment. The graphical values represent the mean gene expression levels relative to the B-actin ± standard error of mean (SEM) (housekeeping gene). Statistical significance between the groups was calculated using a one-way ANOVA followed by Tukey’s multiple comparison test. The results were considered significant from PBS+ unchallenged if *p* < 0.05 * and considered statistically significant from the PBS+ challenged control group if *p* < 0.05 ^~^, in cases of significant statistical difference between two different doses of the same ligand if *p* < 0.05 ^#^.

To measure immune responses, gene expression levels were analyzed in the spleen (Figure 4A–I), cecal tonsils (Figure 5A–I) and lungs (Figure 6A–I). Tissues were collected at 3, 8 and 18 h post TLR treatment and subjected to a real-time PCR to examine the local and systemic immune responses.

Interferon alpha (IFN-α) transcripts (Figure 4A) were upregulated in the spleen at 3 and 18 h post TLR treatment in the high-dose poly(I:C) group, compared to the other treatment groups. Chickens treated with a low dose of CpG ODN 2007 also expressed significant levels of IFN-α at 3 h post-treatment (*p* < 0.05). However, chickens that received the high-dose CpG ODN 2007 treatment showed upregulated transcripts of IFN-α at 8 and 18 h post-treatment (*p* < 0.05). Interferon beta (IFN-ß) expression was significantly downregulated in the low-dose poly(I:C) group at 3 h post TLR treatment (*p* < 0.05). Both the CpG ODN 2007 groups (high and low) upregulated the expression of IFN-ß (Figure 4B) at 8 and 18 h post-treatment. With respect to Interferon gamma (IFN-γ), transcripts were upregulated in both CpG ODN 2007 groups at 3 h post-treatment (*p* < 0.05). Transcripts of IFN-γ (Figure 4C) were upregulated in both the high- and low-dose poly(I:C) groups at 18 h post-treatment (*p* < 0.05), with the low-dose poly(I:C) group upregulated at 8 h post TLR treatment (*p* < 0.05). Additionally, TLR ligands significantly upregulated the level of interferon-stimulated genes (ISGs) in the spleen. The transcripts of viperin in the spleen (Figure 4G) were upregulated in both the CpG ODN 2007 and poly(I:C) groups at 3 and 8 h post-treatment, compared to the other treatment groups. Amongst the two CpG ODN 2007 groups, chickens treated with a low dose of CpG ODN 2007 had higher levels of viperin transcripts at 8 h post-treatment (*p* < 0.05). However, at 18 h, the transcripts in the low-dose CpG ODN 2007 group were downregulated (*p* < 0.05). Similarly, chickens treated with a high and low dose of CpG ODN 2007 reflected upregulated levels of protein kinase R (PKR) transcripts (Figure 4H) at all three time points (*p* < 0.05). The chickens treated with a low dose of CpG ODN 2007 had the highest expression of PKR at 3 h post-treatment. Amongst the two CpG ODN 2007 groups, the low dose of CpG ODN 2007 induced a significantly higher level of PKR transcripts at 3 h post-treatment. Similarly, at 3 and 8 h post-treatment, PKR transcripts were upregulated in chickens treated with high poly(I:C) and low poly(I:C) doses (*p* < 0.05). However, at 18 h post-treatment, transcripts were downregulated in the high- and low-dose poly(I:C) group. Finally, IFITM3 (Figure 4I) expression was upregulated at 8 and 18 h in the high-dose poly(I:C) group. Chickens treated with a low dose of CpG ODN 2007 revealed an upregulated level of IFITM3 at 18 h post-treatment (*p* < 0.05).

Furthermore, CpG ODN 2007 and poly(I:C) induced the expression of pro-inflammatory cytokines in the spleen at different time points. Chickens treated with a low dose of CpG ODN 2007 had a significant upregulation of IL-1ß (Figure 4D) expression at 8 and 18 h post -treatment. High and low doses of poly(I:C) upregulated the expression of IL-1ß at 18 h post TLR treatment (*p* < 0.05). The chickens treated with a low dose of poly(I:C) also upregulated the expression of IL-8 at 3 h post TLR treatment (*p* < 0.05). Both CpG ODN 2007 (high and low) treated groups led to significant upregulation of IL-8 (Figure 4E) expression at 18 h post TLR administration (*p* < 0.05). The expression of IL-18 (Figure 4F) was significantly upregulated at 3 and 8 h post TLR treatment in the chickens of the high-dose poly(I:C) group. Additionally, TLR-treated chickens with the low-dose CpG ODN 2007 group also showed upregulated expression of IL-18 at 8 h post TLR treatment (*p* < 0.05).

##### Cecal Tonsils

TLR ligands CpG ODN 2007 and poly(I:C) induce antiviral responses in cecal tonsils. In cecal tonsils, the expression of IFN-α (Figure 5A) was upregulated in both CpG ODN 2007 and poly(I:C) groups (high and low dose) at 3 h post-treatment (*p* < 0.05). An upregulated expression of IFN-α was observed in the high-dose poly(I:C) group, compared to the low-dose group, at 8 h post-treatment (*p* < 0.05). Chickens treated with a low dose of CpG ODN 2007 exhibited upregulated levels of IFN-α transcripts at 8 and 18 h post-treatment (*p* < 0.05). With respect to IFN-ß, chickens treated with the high or low doses of poly(I:C), significantly upregulated IFN-ß expression at 3 and 8 h post-treatment (Figure 5B). The low dose of CpG ODN 2007 also showed upregulated IFN-ß expression at 8 h post-treatment (*p* < 0.05). A significant downregulation of IFN-ß expression was observed in the high-dose CpG 2007 group at 18 h post-treatment, compared to the other treatment groups. There was a significant difference in the expression of IFN-ß between the CpG ODN 2007 groups at 18 h post-treatment, with the low-dose CpG 2007 group inducing a higher expression of IFN-ß transcripts (*p* < 0.05). Additionally, both the high dose of poly(I:C) and low dose of CpG ODN 2007 significantly induced the expression of IFN-γ (Figure 5C) at 3 and 18 h post-treatment. There was a significant difference in IFN-γ expression between the high and low dose poly(I:C) at 8 h post-treatment, with the chickens treated with a high dose of poly(I:C) showing a higher expression of IFN-γ transcript levels.

TLR ligands also significantly induced the expression of ISGs in cecal tonsils. The low-dose CpG ODN 2007 upregulated the expression of viperin (Figure 5G) at 8 and 18 h post TLR treatment (*p* < 0.05). However, the expression of viperin was downregulated in the high-dose poly(I:C)-treated chickens at 3 h post-treatment. Chickens treated with a low dose of poly(I:C) showed a significant expression of viperin at 18 h post-treatment (*p* < 0.05). Furthermore, chickens treated with a high and low dose of CpG ODN 2007 showed an upregulation of PKR expression (Figure 5H) at 3 h post-treatment. The high poly(I:C) dose significantly induced a higher expression of PKR at 18 h post-treatment (*p* < 0.05). Moreover, the low dose of poly(I:C) and CpG ODN 2007 also significantly upregulated levels of IFITM3 (Figure 5I) at 3 and 8 h post TLR treatment. The treatment of chickens with a high dose of poly(I:C) led to the highest expression of interferon-induced transmembrane protein 3 (IFITM3) at 18 h, with a significant difference from the low-dose poly(I:C)-treated chickens (*p* < 0.05). TLR ligands also induced the expression of different pro-inflammatory cytokines at different time points. Chickens treated with a high dose of poly(I:C) displayed an upregulation of IL-1ß transcripts (Figure 5D) at 3, 8 and 18 h post TLR treatment. The high-dose poly(I:C)-treated chickens showed significant upregulated IL-1ß transcript level compared with low-dose poly(I:C)-treated chickens at all the above-mentioned time points. The high dose of CpG ODN 2007 upregulated the expression of IL-1ß transcript at 3 h post-treatment (*p* < 0.05). Treatment with CpG ODN 2007 (high and low dose) and a high dose of poly(I:C) upregulated the expression of IL-8 (Figure 5E) at 3 and 8 h post-treatment. Chickens treated with a high dose of poly(I:C) also upregulated the expression of IL-8 at 18 h, with a significant difference compared to the low-dose poly(I:C)-treated chickens. The treatment of chickens with a high dose of CpG ODN 2007 led to the highest expression of IL-18 at 3 h post-treatment. The treatment of chickens with a high dose of poly(I:C) induced a significant expression of IL-18 at 18 h post TLR treatment (*p* < 0.05).

##### Lungs

TLR ligands CpG ODN 2007 and poly(I:C) induce antiviral responses in the lungs. The treatment of chickens with a high dose of poly(I:C) resulted in an induction of IFN-α (*p* < 0.05) at 3 and 8 h post-treatment (Figure 6A). Between the two poly(I:C) groups, there was a significant difference in the expression of IFN-α at 3 h post-treatment. Both doses of poly(I:C) along with the high-dose CpG ODN 2007 group significantly enhanced the expression of IFN-α transcripts at 8 h post-treatment (*p* < 0.05). With respect to IFN-ß (Figure 6B), chickens treated with the high and low doses of poly(I:C) had a significantly higher expression of IFN-ß at 3 and 8 h post-treatment. The high dose of poly(I:C) further showed a significantly upregulated expression at 8 and 18 h post-treatment (*p* < 0.05). Additionally, treatment of the chickens with a high dose of poly(I:C) led to significant upregulation of IFN-γ transcripts (Figure 6C) at 3 and 18 h post-treatment. IFN-γ transcripts were also upregulated in chickens treated with a low dose of CpG ODN 2007 at 3 h post-treatment. The transcripts of IFN–γ were significantly downregulated in all treatment (both poly(I:C) and CpG ODN 2007) groups at 8 h post-treatment (*p* < 0.05).

Furthermore, chickens treated with CpG ODN 2007 (high and low dose) and a low dose of poly(I:C) showed a higher expression of IL-1ß (Figure 6D) at 3 h post-treatment. Chickens treated with a low dose of poly(I:C) exhibited a higher expression of IL-1ß expression, compared to the high-dose poly(I:C)-treated chickens at 3 h post TLR treatment (*p* < 0.05). There was also an upregulated expression of IL-1ß in chickens treated with high-dose poly I:C and low-dose CpG ODN 2007 at 8 h post-treatment (*p* < 0.05). Treatment with both doses of poly(I:C) groups induced IL-8 expression (Figure 6E) at 3 and 8 h post TLR treatment. Chickens treated with a low dose of CpG ODN 2007 had an elevated expression of IL-8 transcripts at 3 and 8 h post TLR treatment (*p* < 0.05). The low dose of poly(I:C) upregulated IL-18 (Figure 6F) expression at 3 h post-treatment. The high-dose poly(I:C)-treated chickens, however, showed a significant downregulation in IL-18 expression at 8 h post-treatment. Chickens treated with a low dose of poly(I:C) showed an upregulated expression of IL-18 at 18 h post TLR treatment, compared to the high-dose poly(I:C) group (*p* < 0.05).

##### Administration of CpG ODN 2007 Induces a Higher Level of HI Antibodies Compared to Poly(I:C)

**Figure 7:**
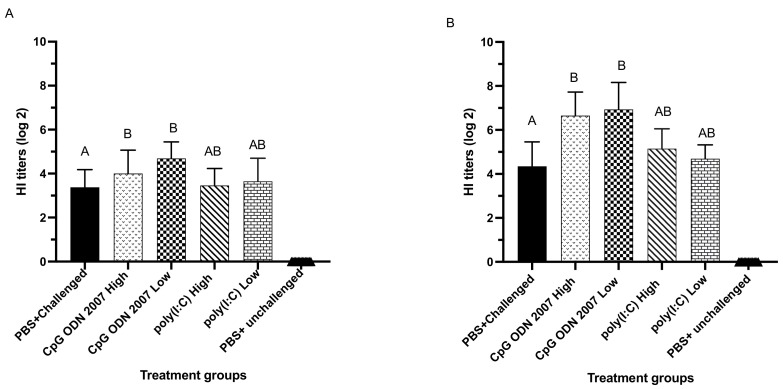
Average serum HI antibody in the seeder chickens of trial 1. On day fourteen of age, the chickens were treated with high and low doses of CpG ODN 2007 and poly(I:C), 18 h prior to H9N2 AIV inoculation. Twenty-four hours later, the seeder chickens were co-housed with recipient chickens. Serum was collected on days 7 (**A**) and 14 PI (**B**). Data were analyzed using a one-way ANOVA followed by Tukey’s multiple comparison test. Group means with the same letter do not differ significantly. The standard error of the mean is indicated with the error bars. The difference between means was considered significant when *p* < 0.05.

Serum samples were collected on days 7 and 14 PI to analyze antibody-mediated responses. The results of HI antibody titers from trial 1 revealed that seeder chickens that were administered with CpG ODN 2007, irrespective of the dosage, had higher HI titers compared to the PBS-challenged group on days 7 and 14 PI (Figure 7). The low dose of CpG ODN 2007 led to a significantly higher induction of antibody titers (4.7 log_2_ scale) on day 7 PI compared to the PBS-challenged group (3.3 log_2_ scale) (Figure 7A). Following day 7 PI, the HI titers in the high- and low-dose CpG ODN 2007 groups were significantly enhanced two-fold on day 14 PI (Figure 7B). The low- and high-dose CpG ODN 2007-treated chickens had significantly higher HI titers compared to the PBS-challenged group, irrespective of the dosage (*p* < 0.05).

## 4. Discussion

H9N2 AIV is a subtype causing outbreaks in the poultry industry. Major concerns associated with this subtype are its high transmission rate, huge economic losses and zoonotic potential. Despite strict biosecurity and preventive measures, H9N2 AIV transmission can still occur in poultry [43]. This increases the risk of outbreaks and raises concerns regarding the effectiveness of these methods. The present study was designed to study the efficacy of TLR ligands to limit H9N2 AIV transmission in a direct contact model in chickens. Our previous studies revealed that TLR ligands can be employed as effective control methods to limit viral replication [37,41]. Previous studies have suggested that TLR ligands can stimulate the immune system and elicit anti-viral responses in chickens [44,45]. The present study thus examined whether the administration of two different doses of TLR ligands (CpG ODN 2007 and poly(I:C)) could interfere with the replication of H9N2 AIV and reduce transmission. The results of the present study revealed that employing TLR ligands can limit virus replication, enhance host anti-viral responses and reduce the transmission of H9N2 AIV from infected to naive recipient chickens. The efficacy of TLR ligands to reduce AIV shedding varied with the dose and nature of the ligand.

Based on the current study, the timing of TLR ligand administration had a considerable impact on the induction of host responses. Our studies previously revealed that the administration of CpG ODN 2007 and poly(I:C), 18 h prior to infection, provided adequate time to initiate innate anti-viral and pro-inflammatory responses which are believed to be involved in a reduction in virus shedding by inhibiting different stages of the H9N2 AIV replication cycle [41]. Our earlier study by St. Paul and colleagues (2012a) suggested that the administration of poly(I:C), CpG and LPS 24 h before AIV infection can reduce virus shedding [36].

The results from trial 1 demonstrated that the intramuscular administration of CpG ODN 2007 or poly(I:C) was effective at reducing oral and cloacal shedding from seeder chickens. The efficacy of these ligands varied with the dosage and type of the ligand. This finding was in agreement with previous studies which demonstrated that the effect of TLRs varies with the type as well as the dosage of the ligand [36,46]. The seeder chickens (trial 1) which were administered with the low dose of CpG ODN 2007 demonstrated a higher reduction in oral virus shedding, compared to chickens in the higher dose group. With respect to poly(I:C) treatment, the higher dose was shown to be relatively effective at reducing oral as well as cloacal shedding, compared to the other treated seeder groups. The results from trial 2 demonstrated that treatment with CpG ODN 2007 and poly(I:C) in the exposed recipient chickens was effective at minimizing H9N2 AIV infection, which occurred via transmission from the contact infected seeder chickens at different time points. Similar to the findings in trial 1, the low dose of CpG ODN 2007 and the high dose of poly(I:C) treatments were shown to be effective in reducing oral and cloacal H9N2 AIV titers from the exposed recipient chickens.

In our previous studies, the reduction in virus shedding in the CpG ODN 2007 and poly(I:C) treated chickens was related to the immunomodulatory nature of the CpG ODN 2007 and poly(I:C) to upregulate the expression of pro-inflammatory and anti-viral cytokines [24,36]. Consistent with the stimulatory nature of class B CpG ODN, we observed the upregulated expression of type I IFNs in the spleen and lungs of the CpG ODN 2007-treated chickens. Previous studies have shown that the administration of CpG ODN 2007 induces IFN-α expression in the spleen and bursa of Fabricius, which further skews the response to T_H 1_ (T helper 1) responses [25,47]. Moreover, our data revealed that immunity conferred in CpG ODN 2007-treated chickens can be correlated with IFN-γ expression in the spleens and cecal tonsils. Our current findings were consistent with our previous studies, which showed that CpG ODN 2007 treatment causes a reduction in viral replication characterized by an enhanced expression of IFN-γ expression in the spleen and bursa of Fabricius [25,48]. CpG ODN 2007 can further differentiate CD4+ T cells towards T_H_1 cells with an enhanced expression of IFN-γ [49,50]. On the other hand, treatment with both doses of poly(I:C) (the seeder chickens in trial 1 and the recipient chickens in trial 2) showed an upregulated expression of type I IFNs in the spleen, cecal tonsil and lungs. A high dose of poly(I:C) (400 ug) was previously shown to upregulate the expression of type I and II IFNs along with pro-inflammatory cytokines, serving as correlates of protection against AIV infections [27,36].

A possible reason for a higher reduction in virus shedding in the high-dose poly(I:C) treated chickens can be attributed to the ability of poly(I:C) to bind to TLR3 and signal via TIR domain-containing adaptor, inducing the IFN (TRIF) and IRF pathway to induce type I and II IFNs in various tissues and cell subsets, such as macrophages and dendritic cells [51,52]. Secondly, the efficiency of the high dose of poly(I:C) to reduce H9N2 AIV shedding can also be attributed to the sensing of poly(I:C) by melanoma differentiation-associated gene 5 (MDA-5), which facilitates the induction of type I IFNs via the utilization of mitochondrial antiviral signaling protein (MAVS) [53]. Thus, it is possible that poly(I:C) utilizes the two downstream pathways that have an additive or synergistic effect on enhancing immune responses and limiting H9N2 AIV replication. The treatment of chickens with a low-dose of poly(I:C) did not result in a significant reduction in the oral and cloacal shedding compared to the other treated groups. This could partly be due to the low concentration of poly(I:C) exposed to in vivo degradation in tissues before binding with the corresponding TLR3 to produce anti-viral effects [54].

With regard to CpG ODN 2007, our previous study highlighted a difference in the dose effect between the high- and low-dose-treated chickens, which can be attributed to the dose-dependent binding of the downstream signaling adaptor molecules such as TRIF Toll/IL-1 receptor (TIR) domain-containing adaptor or TRIF-related adaptor molecule (TRAM) required for the intracellular TLR signaling pathways [41]. This can be supported by a study with human lung epithelial cells, which suggests that TLR ligands activate downstream signaling pathways in a dose-dependent manner [55]. Another possible reason can be the regulation of immune responses via feedback mechanisms. The negative/positive feedback loop machinery was described by Sung and colleagues (2014), in which the expression of a subunit of NF-KB subunit, RelA, was controlled by the transcription factor Ikaros under a specific dose of LPS [56]. A study by Volpi and colleagues (2013) also revealed that a high dose of CpG ODN 1826 can trigger an opposite and tolerogenic response in mouse plasmacytoid dendritic cells in in vivo and in vitro human studies [57].

Our earlier study showed that a low dose of CpG ODN 2007 in chickens induced different expression profiles of ISGs in the spleen, lungs and cecal tonsils. An upregulated expression of IFITM3, PKR and viperin was observed in the lungs and cecal tonsils of the low-dose CpG ODN 2007-treated chickens [41]. This may partly explain the reduced oral and cloacal H9N2 AIV shedding in the low-dose CpG ODN 2007-treated chickens. The induction of ISGs plays an important role in limiting H9N2 AIV replication at different stages, such as cytosolic entry and uncoating, viral protein synthesis and virus budding [58,59,60].

In the current study, treatment with CpG ODN 2007 and poly(I:C) resulted in enhanced pro-inflammatory responses in the spleen, cecal tonsils and lungs. Specifically, there was an upregulated expression of IL-1ß and IL-8 in the spleen, lungs and cecal tonsils of CpG ODN 2007 (high or low) and high-dose poly(I:C)-treated chickens. We only observed an upregulated expression of IL- 18 in the spleen and cecal tonsils of high-dose poly(I:C)-treated chickens. Recent studies have shown that chicken IL-18 has immunomodulatory and anti-viral functions against AIV [61]. Previous studies have shown that IL-1ß can recruit and activate innate cell subsets and act as a main indicator for macrophage activation [62]. This cytokine can also facilitate antibody-mediated immune responses by inducing specific Th17-mediated responses [62,63]. IL-1ß increases vascular permeability and induces the production of adhesion molecules and cytokines such as IL-6, IL-8, IL-17 and IFN-γ. This could be associated with the upregulated expression of IL-8 in the lungs. IL-8 expression in the lungs raises the possibility of the migration of heterophils to the site of virus replication. Heterophils have previously been shown to have phagocytotic activity as an early innate mechanism to reduce infection via the secretion of anti-microbial peptides or production of cytokines [64,65].

Our results are in agreement with those of previous studies which demonstrated that CpG ODN can lead to a reduction in H4N6 AIV replication by the induction of IL-1ß, IFN-γ and NO (nitric oxide) production in vitro [41,47,66]. The treatment of chickens with poly(I:C) in vivo can decrease H4N6 AIV load in tissues by upregulating pro-inflammatory and anti-viral cytokines: IL-1ß, IL-6, IL-8, 1L-18, IFN-γ and NO production [20,33,46]. There was no significant difference in the expression of different immune system genes between the PBS+ challenged and the PBS+ unchallenged chickens in the spleen, cecal tonsils and lungs. This can be related to the selective nature of the AIVs to replicate in the mucosal sites rather than being systemic in nature. This finding is in alignment with a previous study by Mahana and colleagues (2019), which showed that H9N2 AIV can effectively infect chickens; however, the level of gene expression in the spleen and lung was not significant compared to the uninfected control chickens [67]. This observation was in alignment with the findings of Cao and colleagues (2017) that the induction of innate responses in chickens varies with the subtype of Influenza A viruses and their site of infection [68]. Previous work from our lab also did not show a difference between the expression of these genes in cecal tonsils in the first 24 hours of H9N2 AIV inoculation [69].

The results presented here indicate that the transmission of H9N2 AIV can occur via direct contact in chickens. It has previously been reported that AIV infection can occur between chickens when kept in direct contact [70,71]. The results from experiment 1 revealed that there was reduced oral and cloacal shedding from the recipient chickens that were exposed to CpG ODN 2007 and poly(I:C)-treated seeder chickens. The recipient chickens that were exposed to the low-dose CpG ODN 2007-treated seeder chickens showed the highest reduction in virus shedding titers, followed by the recipient chickens exposed to the high-dose poly(I:C) seeder group. The low dose of CpG ODN 2007 and high-dose poly(I:C) groups saw a smaller number of recipient chickens positive in H9N2 AIV isolation at each time point. This can be attributed to the findings from our study that highlight the anti-viral and pro-inflammatory functions of CpG ODN 2007 and poly(I:C) ligands that may have interfered with the replication of H9N2 AIV in seeder chickens, leading to lower virus shedding through the oral and cloacal routes [41]. Hence, it is reasonable to speculate that due to overall decreased H9N2 AIV shedding and a relatively low number of seeder chickens positive in virus isolation, there might have been less virus dissemination and decreased chances of contact-based transmission of H9N2 AIV from treated seeder to exposed recipient chickens.

Similarly, in trial 2, the results demonstrated that recipient chickens that received CpG ODN 2007 and poly(I:C) treatment can be infected with H9N2 AIV when kept in direct contact with inoculated seeder chickens. However, the rate of infection in the recipient chickens was observed to be reduced in terms of virus shedding and the number of infected chickens. We observed a reduced magnitude of oral and cloacal shedding from low-dose CpG ODN 2007 and high-dose poly(I:C)-treated exposed recipient chickens. This can be attributed to the immunostimulatory nature of CpG ODN 2007 and poly(I:C) ligands to trigger anti-viral and pro-inflammatory responses which minimize virus replication [25,44,72]. The innate responses induced by the administration of these ligands enhance host immunity against different invading pathogens and facilitate cross talk mechanisms between the innate and antibody-mediated immune responses in chickens [51,71,73,74]. Thus, it can be concluded that the CpG ODN 2007 and poly(I:C) were effective at inducing protective immune responses in exposed recipient chickens that helped in reducing H9N2 AIV infection, which would have occurred via transmission from the inoculated seeder chickens.

In conclusion, the results from the current study revealed that TLR ligands as stand-alone agents can be employed as effective control methods for limiting H9N2 AIV replication in chickens. Being immunostimulatory in nature, treatment with poly(I:C) and CpG ODN 2007 led to enhanced innate responses characterized by an upregulated expression of type I and II IFNs, pro-inflammatory cytokines and ISGs in the spleen, cecal tonsils and lungs. These findings suggest that TLR ligands play an integral role in controlling AIV infections. Further studies should focus on exploring combinations of poly(I:C) and CpG ODN 2007 for their direct anti-viral effects or as vaccine adjuvants. Moreover, future studies should explore the potential of TLR ligands in limiting transmission in various AIV transmission models focusing on respiratory, fomite or fecal routes of transmission.

## Figures and Tables

**Table 1:** Primer sequences used for quantitative real-time polymerase chain reaction.

Gene	Primer Sequence	Annealing Temperature	References
β-actin	F:5′-CAACACAGTGCTGTCTGGTGGTA-3′ R: 5′-ATCGTACTCCTGCTTGCTGATCC-3′	58	[25]
IFN-γ	F: 5′-ACA CTG ACA AGT CAA AGC CGC ACA-3′ R: 5′-AGT CGT TCA TCG GGA GCT TGG C-3′	60	[39]
IFN-α	F: 5′-ATCCTGCTGCTCACGCTCCTTCT-3′ R: 5′-GGTGTTGCTGGTGTCCAGGATG-3′	64	[25]
IFN-β	F: 5′-GCCTCCAGCTCCTTCAGAATACG-3′ R: 5′-CTGGATCTGGTTGAGGAGGCTGT-3′	64	[40]
PKR	F: 5′-TGGTACAGGCGTTGGTAAGAG-3′ R: 5′-GAGCACATCCGCAGGTAGAG-3′	60	[41]
IFITM3	F: 5′-CACACCAGCATCAACATGCC-3′ R: 5′-CCTACGAAGTCCTTGGCGAT-3′	60	[41]
Viperin	F: 5′-GGAGGCGGGAATGGAGAAAA-3′ R: 5′-CAGCTGGCCTACAAATTCGC-3′	60	[41]
IL-8	F:5′- CCAAGCACACCTCTCTTCCA-3′ R:5′- GCAAGGTAGGACGCTGGTAA-3′	64	[25]
IL-18	F:5′-GAAACGTCAATAGCCAGTTGC-3′ R:5′-TCCCATGCTCTTTCTCACAACA-3′	64	[42]
IL-1β	F:5’-GTGAGGCTCAACATTGCGCTGTA-3’	60	[40]
	R:5’-TGTCCAGGCGGTAGAAGATGAAG-3’		

IFN, interferon; PKR, protein kinase R; IFITM3, interferon-induced transmembrane protein 3.

**Table 2:** Virus isolation from TLR-treated seeder chickens of different treatment groups (*n* = 16).

Oral Swabs PI (Days)	CpG ODN 2007 High	CpG ODN 2007 Low	Poly(I:C) High	Poly(I:C) Low	PBS + Infec
3	15/16 (93%)	13/16 (81%)	13/16 (81%)	14/16 (88%)	16/16 (100%)
5	13/16 (81%)	11/16 (68%)	12/16 (75%)	12/16 (75%)	14/16 (88%)
7	11/16 (68%)	8/16 (50%)	7/16 (43%)	9/16 (56%)	11/16 (68%)
9	5/16 (31%)	3/16 (18%)	0/16 (0%)	7/16 (43%)	8/16 (50%)
**Cloacal swabs**	**CpG ODN 2007** **High**	**CpG ODN 2007** **Low**	**poly(I:C) High**	**poly(I:C)** **Low**	**PBS + infec**
3	15/16 (93%)	10/16 (62%)	12/16 (75%)	14/16 (88%)	16/16 (100%)
5	14/16 (88%)	9/16 (56%)	11/16 (68%)	13/16 (81%)	14/16 (88%)
7	13/16 (81%)	8/16 (50%)	9/16 (56%)	11/16 (68%)	12/16 (75%)
9	4/16 (25%)	2/16 (12%)	0/16 (0%)	4/16 (25%)	8/16 (50%)

No. of swabs positive/no. of swabs tested.

**Table 3:** Rate of infection in recipient chickens of different treatment groups (*n* = 8).

Oral Swabs PI (Days)	CpG ODN 2007 High	CpG ODN 2007 Low	Poly(I:C) High	Poly(I:C) Low	PBS
3	6/8 (75%)	4/8 (50%)	7/8 (87%)	5/8 (62%)	7/8 (87%)
5	5/8 (62%)	4/8 (50%)	6/8 (75%)	3/8 (38%)	7/8 (87%)
7	5/8 (62%)	1/8 (12%)	5/8 (62%)	3/8 (38%)	5/8 (62%)
9	0/8 (0%)	0/8 (0%)	0/8 (0%)	0/8 (0%)	0/8 (0%)
**Cloacal swabs**	**CpG** **ODN 2007 High**	**CpG ODN 2007 Low**	**poly(I:C)** **High**	**poly(I:C)** **Low**	**PBS + infec**
3	7/8 (87%)	3/8 (38%)	4/8 (50%)	6/8 (75%)	7/8 (87%)
5	7/8 (87%)	0/8 (0%)	2/8 (25%)	6/8 (75%)	7/8 (87%)
7	6/8 (75%)	0/8 (0%)	0/8 (0%)	5/8 (62%)	6/8 (75%)
9	0/8 (0%)	0/8 (0%)	0/8 (0%)	0/8 (0%)	0/8 (0%)

No. of swabs positive/no. of swabs tested.

**Table 4:** Rate of infection in recipient chickens of different treatment groups.

Oral Swabs PI (Days)	CpG ODN 2007 High	CpG ODN 2007 Low	Poly(I:C) High	Poly(I:C) Low	PBS
3	6	3	2	6	7
5	5	3	2	6	6
7	4	1	1	4	6
9	0	0	0	0	0
**Cloacal swabs**	**CpG** **ODN 2007 High**	**CpG ODN 2007 Low**	**poly(I:C)** **High**	**poly(I:C)** **Low**	**PBS**
3	6	2	2	6	7
5	5	2	2	6	6
7	4	1	1	4	6
9	0	0	0	0	0

No. of swabs positive/no. Of swabs tested.

## Data Availability

Not applicable.

## Supplementary Materials

The following supporting information can be downloaded at: https://www.mdpi.com/article/10.3390/v15010238/s1, Figure S1: Experimental design for Trial 1, Figure S2: Experimental design for Trial 2, Figure S3: The figure represents the H9N2 AIV shedding (expressed in TCID_50_/mL) in the oral (A–D) and cloacal swabs (D–G) on days 3-, 5-, 7,- and 9 PI in the seeder chickens of trial 2.

## References

[1] Bouvier N.M., Palese P. (2008). The biology of influenza viruses. Vaccine.

[2] Alexander D.J. (2000). A review of avian in¯uenza in different bird species. Vet. Microbiol..

[3] Suarez D.L. (2010). Avian influenza: Our current understanding. Anim. Health Res. Rev..

[4] Cáceres C.J., Rajao D.S., Perez D.R. (2021). Airborne Transmission of Avian Origin H9N2 Influenza A Viruses in Mammals. Viruses.

[5] Li X., Shi J., Guo J., Deng G., Zhang Q., Wang J., He X., Wang K., Chen J., Li Y. (2014). Genetics, Receptor Binding Property, and Transmissibility in Mammals of Naturally Isolated H9N2 Avian Influenza Viruses. PLoS Pathog..

[6] Guo J., Wang Y., Zhao C., Gao X., Zhang Y., Li J., Wang M., Zhang H., Liu W., Wang C. (2021). Molecular characterization, receptor binding property, and replication in chickens and mice of H9N2 avian influenza viruses isolated from chickens, peafowls, and wild birds in eastern China. Emerg. Microbes Infect..

[7] Nagy A., Mettenleiter T.C., Abdelwhab E.M. (2017). A brief summary of the epidemiology and genetic relatedness of avian influenza H9N2 virus in birds and mammals in the Middle East and North Africa. Epidemiol. Infect..

[8] Thuy D.M., Peacock T.P., Bich V.T.N., Fabrizio T., Hoang D.N., Tho N.D., Diep N.T., Nguyen M., Hoa L.N.M., Trang H.T.T. (2016). Prevalence and diversity of H9N2 avian influenza in chickens of Northern Vietnam, 2014. Infect. Genet. Evol..

[9] Peacock T.P., James J., Sealy J.E., Iqbal M. (2019). A Global Perspective on H9N2 Avian Influenza Virus. Viruses.

[10] Zhou J., Wu J., Zeng X., Huang G., Zou L., Song Y., Gopinath D., Zhang X., Kang M., Lin J. (2016). Isolation of H5N6, H7N9 and H9N2 avian influenza A viruses from air sampled at live poultry markets in China, 2014 and 2015. Eurosurveillance.

[11] Ferro P.J., Budke C.M., Peterson M.J., Cox D., Roltsch E., Merendino T., Nelson M., Lupiani B. (2010). Multiyear Surveillance for Avian Influenza Virus in Waterfowl from Wintering Grounds, Texas Coast, USA. Emerg. Infect. Dis..

[12] Yassine H.M., Lee C.-W., Gourapura R., Saif Y.M. (2010). Interspecies and intraspecies transmission of influenza A viruses: Viral, host and environmental factors. Anim. Health Res. Rev..

[13] Kye S.-J., Park M.-J., Kim N.-Y., Lee Y.-N., Heo G.-B., Baek Y.-K., Shin J.-I., Lee M.-H., Lee Y.-J. (2021). Pathogenicity of H9N2 low pathogenic avian influenza viruses of different lineages isolated from live bird markets tested in three animal models: SPF chickens, Korean native chickens, and ducks. Poult. Sci..

[14] Iqbal M., Yaqub T., Mukhtar N., Shabbir M.Z., McCauley J.W. (2013). Infectivity and transmissibility of H9N2 avian influenza virus in chickens and wild terrestrial birds. Vet. Res..

[15] Saenz R.A., Essen S.C., Brookes S.M., Iqbal M., Wood J.L.N., Grenfell B.T., McCauley J.W., Brown I.H., Gog J.R. (2012). Quantifying Transmission of Highly Pathogenic and Low Pathogenicity H7N1 Avian Influenza in Turkeys. PLoS ONE.

[16] Wang J., Wu M., Hong W., Fan X., Chen R., Zheng Z., Zeng Y., Huang R., Zhang Y., Lam T.T.-Y. (2016). Infectivity and Transmissibility of Avian H9N2 Influenza Viruses in Pigs. J. Virol..

[17] World Health Organization (2011). Manual for the Laboratory Diagnosis and Virological Surveillance of Influenza.

[18] Zhang G., Xu L., Zhang J., Fang Q., Zeng J., Liu Y., Ke C. (2022). A H9N2 Human Case and Surveillance of Avian Influenza Viruses in Live Poultry Markets—Huizhou City, Guangdong Province, China, 2021. China CDC Wkly..

[19] Alqazlan N., Astill J., Raj S., Sharif S. (2022). Strategies for enhancing immunity against avian influenza virus in chickens: A review. Avian Pathol..

[20] Kapczynski D.R., Swayne D.E., Compans W., Orenstein W.A. (2009). Influenza Vaccines for Avian Species. Vaccines for Pandemic Influenza.

[21] Singh S.M., Alkie T.N., Hodgins D.C., Nagy É., Shojadoost B., Sharif S. (2015). Systemic immune responses to an inactivated, whole H9N2 avian influenza virus vaccine using class B CpG oligonucleotides in chickens. Vaccine.

[22] Van der Goot J.A., Koch G., de Jong M.C.M., van Boven M. (2005). Quantification of the effect of vaccination on transmission of avian influenza (H7N7) in chickens. Proc. Natl. Acad. Sci. USA..

[23] Abdul-Cader M.S., Ahmed-Hassan H., Amarasinghe A., Nagy E., Sharif S., Abdul-Careem M.F. (2017). Toll-like receptor (TLR)21 signalling-mediated antiviral response against avian influenza virus infection correlates with macrophage recruitment and nitric oxide production. J. Gen. Virol..

[24] Barjesteh N., Behboudi S., Brisbin J.T., Villanueva A.I., Nagy E., Sharif S. (2014). TLR ligands induce antiviral responses in chicken macrophages. PLoS ONE.

[25] St Paul M., Mallick A.I., Haq K., Orouji S., Abdul-Careem M.F., Sharif S. (2011). In vivo administration of ligands for chicken toll-like receptors 4 and 21 induces the expression of immune system genes in the spleen. Vet. Immunol. Immunopathol..

[26] Brownlie R., Allan B. (2011). Avian toll-like receptors. Cell Tissue Res..

[27] Karpala A.J., Lowenthal J.W., Bean A.G. (2008). Activation of the TLR3 pathway regulates IFNβ production in chickens. Dev. Comp. Immunol..

[28] St. Paul M., Brisbin J.T., Abdul-Careem M.F., Sharif S. (2013). Immunostimulatory properties of Toll-like receptor ligands in chickens. Vet. Immunol. Immunopathol..

[29] Temperley N.D., Berlin S., Paton I.R., Griffin D.K., Burt D.W. (2008). Evolution of the chicken Toll-like receptor gene family: A story of gene gain and gene loss. BMC Genom..

[30] Akira S., Uematsu S., Takeuchi O. (2006). Pathogen Recognition and Innate Immunity. Cell.

[31] Bavananthasivam J., Alkie T.N., Matsuyama-Kato A., Hodgins D.C., Sharif S. (2019). Characterization of innate responses induced by in ovo administration of encapsulated and free forms of ligands of Toll-like receptor 4 and 21 in chicken embryos. Res. Vet. Sci..

[32] Bavananthasivam J., Kulkarni R.R., Read L., Sharif S. (2018). Reduction of Marek’s Disease Virus Infection by Toll-Like Receptor Ligands in Chicken Embryo Fibroblast Cells. Viral Immunol..

[33] Dar A., Potter A., Tikoo S., Gerdts V., Lai K., Babiuk L.A., Mutwiri G. (2009). CpG Oligodeoxynucleotides Activate Innate Immune Response that Suppresses Infectious Bronchitis Virus Replication in Chicken Embryos. Avian Dis..

[34] Kannaki T.R., Reddy M.R., Shanmugam M., Verma P.C., Sharma R.P. (2010). Chicken toll-like receptors and their role in immunity. World’s Poult. Sci. J..

[35] Hopkins P.A., Sriskandan S. (2005). Mammalian Toll-like receptors: To immunity and beyond. Clin. Exp. Immunol..

[36] St. Paul M., Mallick A.I., Read L.R., Villanueva A.I., Parvizi P., Abdul-Careem M.F., Nagy É., Sharif S. (2012). Prophylactic treatment with Toll-like receptor ligands enhances host immunity to avian influenza virus in chickens. Vaccine.

[37] Barjesteh N., Alkie T.N., Hodgins D.C., Nagy É., Sharif S. (2016). Local Innate Responses to TLR Ligands in the Chicken Trachea. Viruses.

[38] Reed L.J., Muench H. (1938). A simple method of estimating fifty per cent endpoints. Am. J. Epidemiol..

[39] Brisbin J.T., Gong J., Parvizi P., Sharif S. (2010). Effects of Lactobacilli on Cytokine Expression by Chicken Spleen and Cecal Tonsil Cells. Clin. Vaccine Immunol. CVI.

[40] Villanueva A.I., Kulkarni R.R., Sharif S. (2011). Synthetic double-stranded RNA oligonucleotides are immunostimulatory for chicken spleen cells. Dev. Comp. Immunol..

[41] Barjesteh N., Shojadoost B., Brisbin J.T., Emam M., Hodgins D.C., Nagy É., Sharif S. (2015). Reduction of avian influenza virus shedding by administration of Toll-like receptor ligands to chickens. Vaccine.

[42] Brisbin J.T., Zhou H., Gong J., Sabour P., Akbari M.R., Haghighi H.R., Yu H., Clarke A., Sarson A.J., Sharif S. (2008). Gene expression profiling of chicken lymphoid cells after treatment with Lactobacillus acidophilus cellular components. Dev. Comp. Immunol..

[43] Capua I., Terregino C., Cattoli G., Mutinelli F., Rodriguez J.F. (2003). Development of a DIVA (Differentiating Infected from Vaccinated Animals) strategy using a vaccine containing a heterologous neuraminidase for the control of avian influenza. Avian Pathol..

[44] Barjesteh N., O’Dowd K., Vahedi S.M. (2020). Antiviral responses against chicken respiratory infections: Focus on avian influenza virus and infectious bronchitis virus. Cytokine.

[45] Engel A.L., Holt G.E., Lu H. (2011). The pharmacokinetics of Toll-like receptor agonists and the impact on the immune system. Expert Rev. Clin. Pharmacol..

[46] Kimani F.W., Ajit J., Galluppi A., Manna S., Howitz W.J., Tang S., Esser-Kahn A.P. (2021). Receptor-Ligand kinetics influence the mechanism of action of covalently linked TLR ligands. ACS Chem. Biol..

[47] Patel B.A., Gomis S., Dar A., Willson P.J., Babiuk L.A., Potter A., Mutwiri G., Tikoo S.K. (2008). Oligodeoxynucleotides containing CpG motifs (CpG-ODN) predominantly induce Th1-type immune response in neonatal chicks. Dev. Comp. Immunol..

[48] St. Paul M., Paolucci S., Read L.R., Sharif S. (2012). Characterization of responses elicited by Toll-like receptor agonists in cells of the bursa of Fabricius in chickens. Vet. Immunol. Immunopathol..

[49] Barjesteh N., Brisbin J.T., Behboudi S., Nagy É., Sharif S. (2015). Induction of Antiviral Responses Against Avian Influenza Virus in Embryonated Chicken Eggs with Toll-Like Receptor Ligands. Viral Immunol..

[50] Ciraci C., Lamont S.J. (2011). Avian-specific TLRs and downstream effector responses to CpG-induction in chicken macrophages. Dev. Comp. Immunol..

[51] Matsumoto M., Funami K., Oshiumi H., Seya T. (2004). Toll-Like Receptor 3: A Link between Toll-Like Receptor, Interferon and Viruses. Microbiol. Immunol..

[52] Matsumoto M., Seya T. (2008). TLR3: Interferon induction by double-stranded RNA including poly(I:C). Adv. Drug Deliv. Rev..

[53] Hayashi T., Watanabe C., Suzuki Y., Tanikawa T., Uchida Y., Saito T. (2014). Chicken MDA5 Senses Short Double-Stranded RNA with Implications for Antiviral Response against Avian Influenza Viruses in Chicken. J. Innate Immun..

[54] Bayyurt B., Tincer G., Almacioglu K., Alpdundar E., Gursel M., Gursel I. (2017). Encapsulation of two different TLR ligands into liposomes confer protective immunity and prevent tumor development. J. Control Release.

[55] Thaikoottathil J., Chu H.W. (2011). MAPK/AP-1 activation mediates TLR2 agonist-induced SPLUNC1 expression in human lung epithelial cells. Mol. Immunol..

[56] Sung M.-H., Li N., Lao Q., Gottschalk R.A., Hager G.L., Fraser I.D.C. (2014). Switching of the Relative Dominance between Feedback Mechanisms in Lipopolysaccharide-Induced NF-κB Signaling. Sci. Signal..

[57] Volpi C., Fallarino F., Pallotta M.T., Bianchi R., Vacca C., Belladonna M.L., Orabona C., DE Luca A., Boon L., Romani L. (2013). High doses of CpG oligodeoxynucleotides stimulate a tolerogenic TLR9–TRIF pathway. Nat. Commun..

[58] Goossens K.E., Ward A.C., Lowenthal J.W., Bean A.G. (2013). Chicken interferons, their receptors and interferon-stimulated genes. Dev. Comp. Immunol..

[59] Ivashkiv L.B., Donlin L.T. (2014). Regulation of type I interferon responses. Nat. Rev. Immunol..

[60] Zhou X., Michal J.J., Zhang L., Ding B., Lunney J.K., Liu B., Jiang Z. (2013). Interferon Induced IFIT Family Genes in Host Antiviral Defense. Int. J. Biol. Sci..

[61] Rahman M.M., Uyangaa E., Han Y.W., Kim S.B., Kim J.H., Choi J.Y., Eo S.K. (2012). Enhancement of Th1-biased protective immunity against avian influenza H9N2 virus via oral co-administration of attenuated Salmonella enterica serovar Typhimurium expressing chicken interferon-α and interleukin-18 along with an inactivated vaccine. BMC Vet. Res..

[62] Hebel K., Rudolph M., Kosak B., Chang H.-D., Butzmann J., Brunner-Weinzierl M.C. (2011). IL-1β and TGF-β Act Antagonistically in Induction and Differentially in Propagation of Human Proinflammatory Precursor CD4+ T Cells. J. Immunol..

[63] Puhlmann M., Weinreich D.M., Farma J.M., Carroll N.M., Turner E.M., Alexander H.R. (2005). Interleukin-1β induced vascular permeability is dependent on induction of endothelial Tissue Factor (TF) activity. J. Transl. Med..

[64] Maxwell M.H., Robertson G.W. (1998). The avian heterophil leucocyte: A review. World’s Poult. Sci. J..

[65] Guriec N., Bussy F., Gouin C., Mathiaud O., Quero B., Le Goff M., Collén P.N. (2018). Ulvan Activates Chicken Heterophils and Monocytes through Toll-Like Receptor 2 and Toll-Like Receptor 4. Front. Immunol..

[66] Xie H., Raybourne R.B., Babu U.S., Lillehoj H.S., Heckert R.A. (2003). CpG-induced immunomodulation and intracellular bacterial killing in a chicken macrophage cell line. Dev. Comp. Immunol..

[67] Mahana O., Arafa A.-S., Erfan A., Hussein H.A., Shalaby M.A. (2019). Pathological changes, shedding pattern and cytokines responses in chicks infected with avian influenza-H9N2 and/or infectious bronchitis viruses. Virus Dis..

[68] Cao Y., Huang Y., Xu K., Liu Y., Li X., Xu Y., Zhong W., Hao P. (2017). Differential responses of innate immunity triggered by different subtypes of influenza a viruses in human and avian hosts. BMC Med. Genom..

[69] Alqazlan N., Emam M., Nagy E., Bridle B., Sargolzaei M., Sharif S. (2021). Transcriptomics of chicken cecal tonsils and intestine after infection with low pathogenic avian influenza virus H9N2. Sci. Rep..

[70] Ku K.B., Park E.H., Yum J., Kim H.M., Kang Y.M., Kim J.C., Kim J.A., Kim H.S., Seo S.H. (2014). Transmissibility of novel H7N9 and H9N2 avian influenza viruses between chickens and ferrets. Virology.

[71] Raj S., Astill J., Alqazlan N., Boodhoo N., Hodgins D.C., Nagy É., Mubareka S., Karimi K., Sharif S. (2022). Transmission of H9N2 Low Pathogenicity Avian Influenza Virus (LPAIV) in a Challenge-Transmission Model. Vaccines.

[72] Akira S., Takeda K. (2004). Toll-like receptor signalling. Nat. Rev. Immunol..

[73] Brownlie R., Zhu J., Allan B., Mutwiri G.K., Babiuk L.A., Potter A., Griebel P. (2009). Chicken TLR21 acts as a functional homologue to mammalian TLR9 in the recognition of CpG oligodeoxynucleotides. Mol. Immunol..

[74] Takaoka A., Yanai H. (2006). Interferon signalling network in innate defence. Cell Microbiol..

