# Supplementary figures and images for: Determining the Protective Efficacy of Toll-Like Receptor Ligands to Minimize H9N2 Avian Influenza Virus Transmission in Chickens
Source: Viruses. 2023 Jan 14;15(1):238. doi: 10.3390/v15010238 (PMC9861619; doi:10.3390/v15010238)

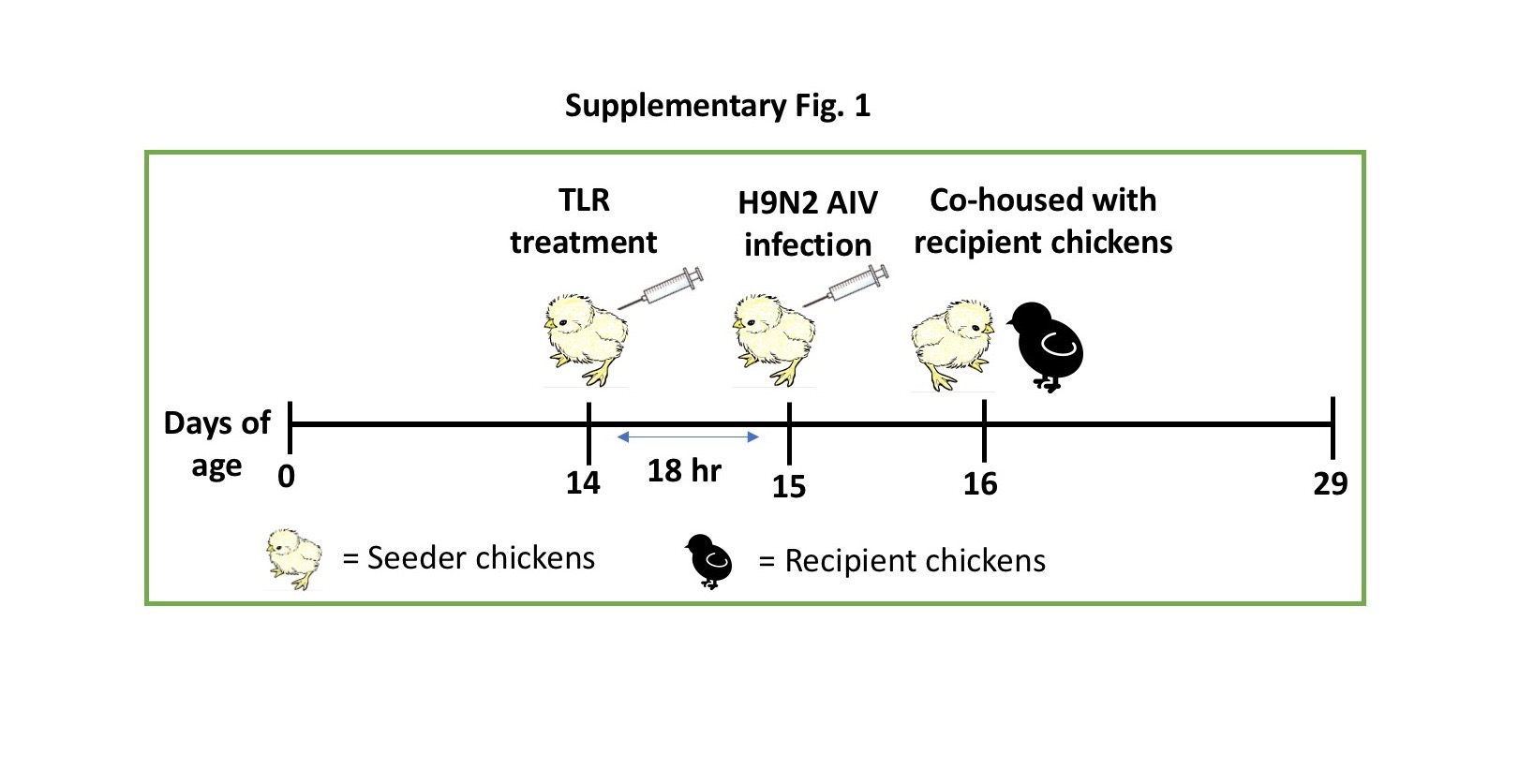

Supplement: Supplementary file 1 [file viruses-15-00238-s001.zip › supplementary figures/Supplementary figure 1.jpg]

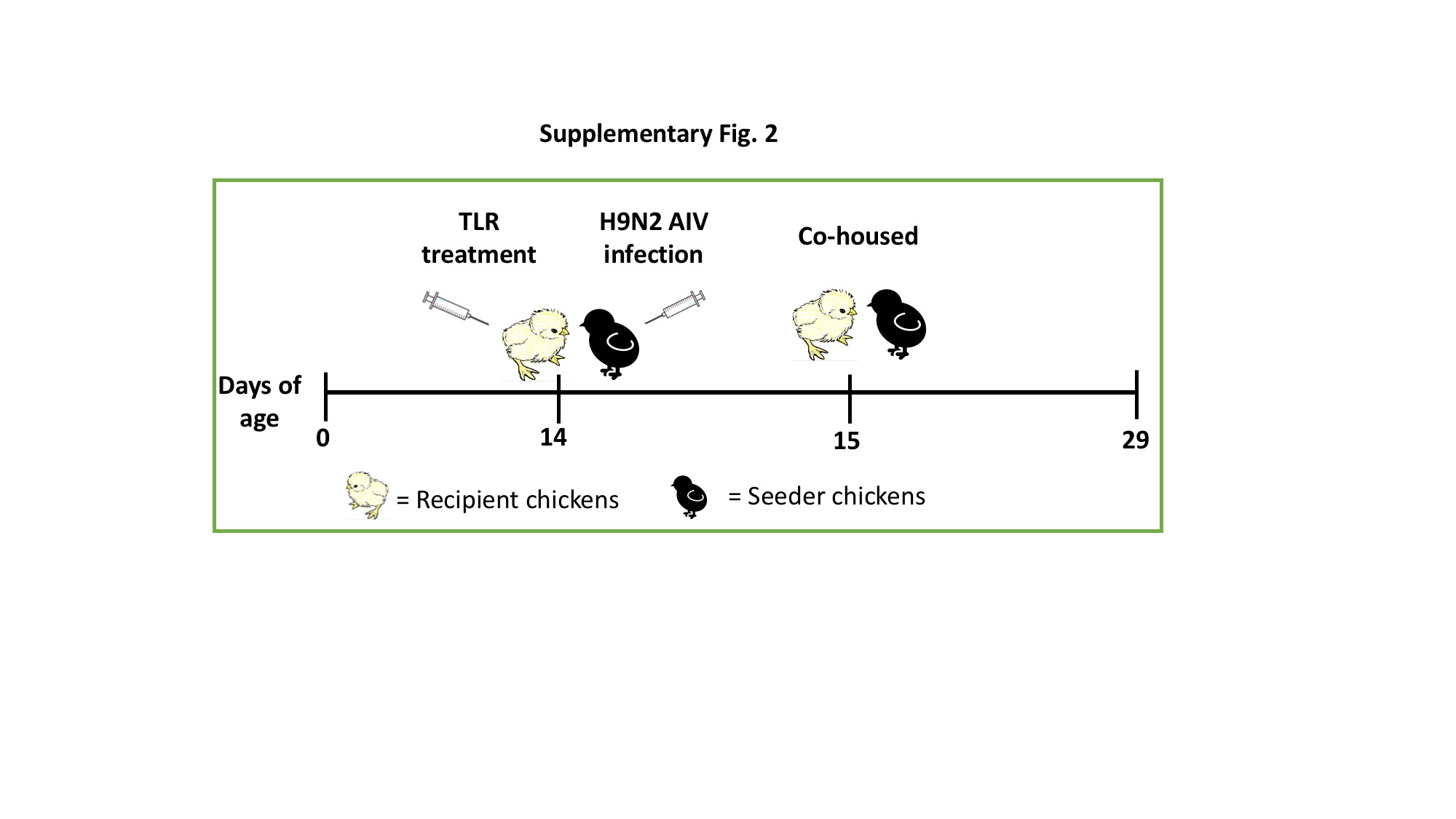

Supplement: Supplementary file 1 [file viruses-15-00238-s001.zip › supplementary figures/Supplementary Figure 2.jpg]

Supplementary Fig. 3

Oral Shedding

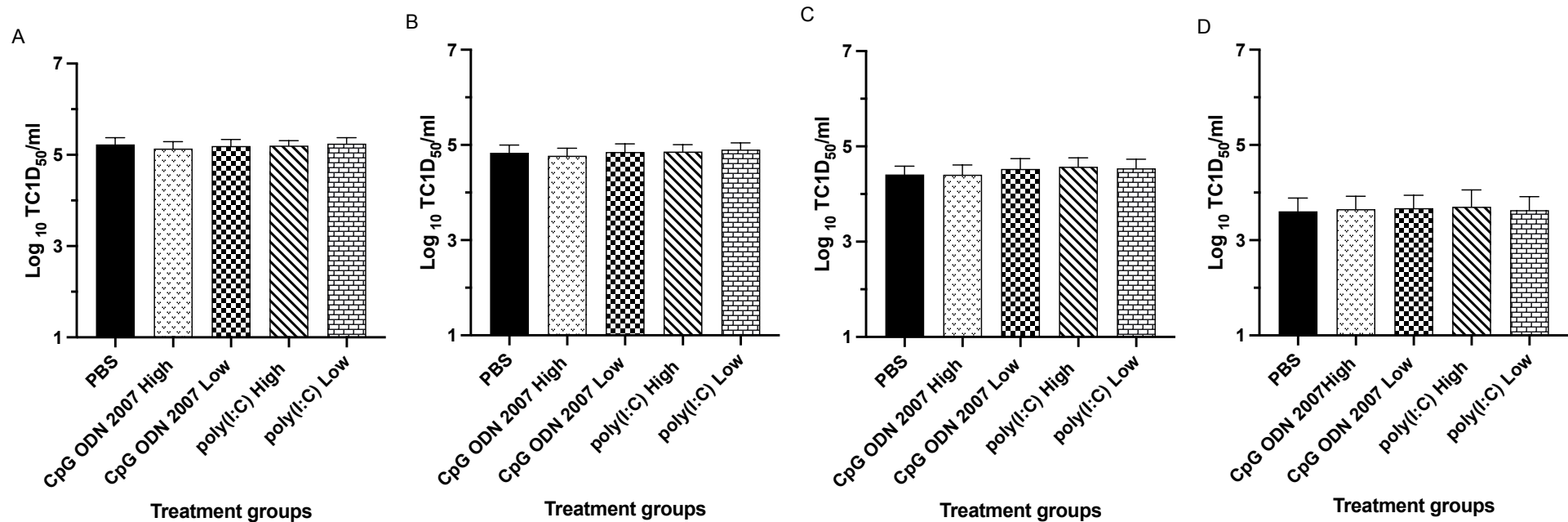

Cloacal Shedding

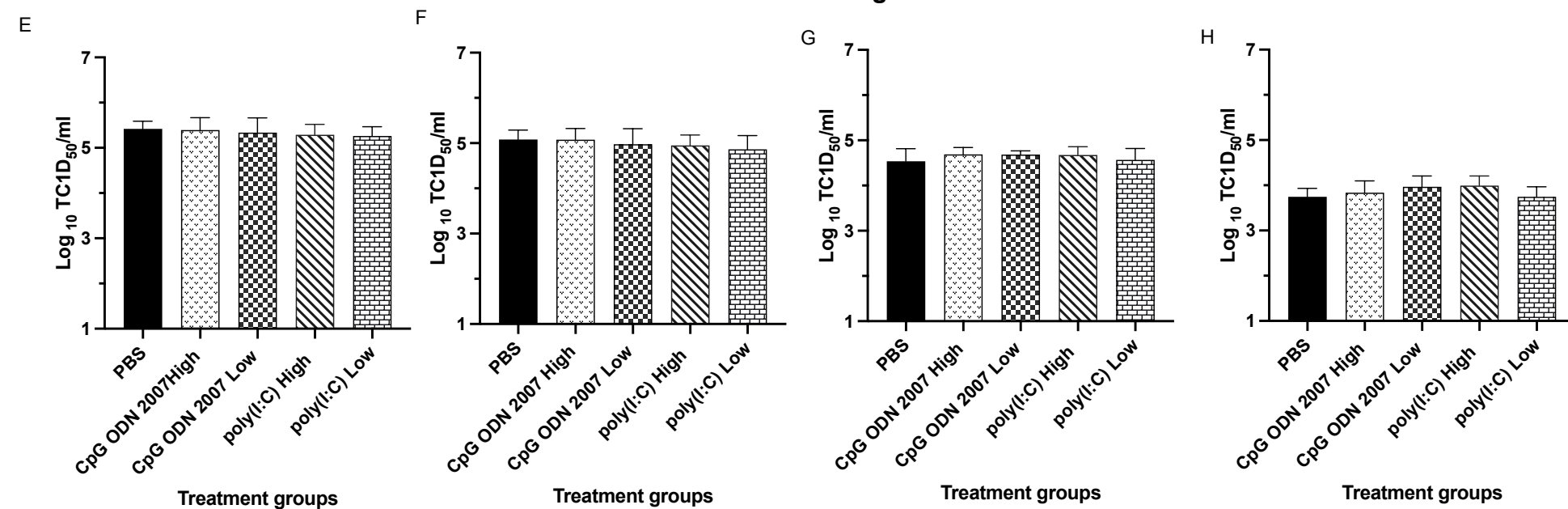

Supplement: Supplementary file 1 [file viruses-15-00238-s001.zip › supplementary figures/supplementary figure 3. Dec 2022.pdf]
